# Why Does Positioning the Scrub Nurse on the Patient's Left Side Enhance Safety and Efficiency in Cardiac Surgery?

**DOI:** 10.7759/cureus.85529

**Published:** 2025-06-07

**Authors:** Idriss Souko, Sulyman Abraheem

**Affiliations:** 1 Cardiothoracic Surgery, Dubai Hospital, Dubai, ARE; 2 Pharmacology, University of Benghazi, Benghazi, LBY

**Keywords:** cardiac surgery, ergonomics, operating room efficiency, scrub nurse positioning, visual access

## Abstract

In cardiac surgery, optimal operating room (OR) dynamics are essential for patient safety and procedural efficiency. This article highlights the impact of positioning the scrub nurse on the patient’s left side, opposite the primary surgeon, on surgical workflow and outcomes. Enhanced direct visual access to the operative field enables the scrub nurse to anticipate needs, improve communication, and reduce verbal prompts. Ergonomically, this position shortens instrument hand-off pathways, minimizing delays and errors. Coordination with the assistant surgeon is streamlined, further promoting seamless teamwork. Supported by human factors research and personal clinical experience, this configuration demonstrates measurable improvements in safety, efficiency, and team dynamics. Reevaluating traditional scrub nurse placement can thus contribute significantly to optimized cardiac surgery practice.

## Editorial

In cardiac surgery's complex, high-stakes environment, every movement, position, and role in the operating room (OR) contributes to patient safety, procedural timing, and outcomes. Among these elements, the positioning of the scrub nurse, often considered traditional or fixed, warrants reevaluation through the lens of human factors, workflow dynamics, and spatial ergonomics. Specifically, placing the scrub nurse on the patient’s left side (opposite the primary surgeon) enhances visual access, communication, and instrument delivery, with measurable impacts on surgical performance.

Direct visual access: a critical advantage

**Figure 1:**
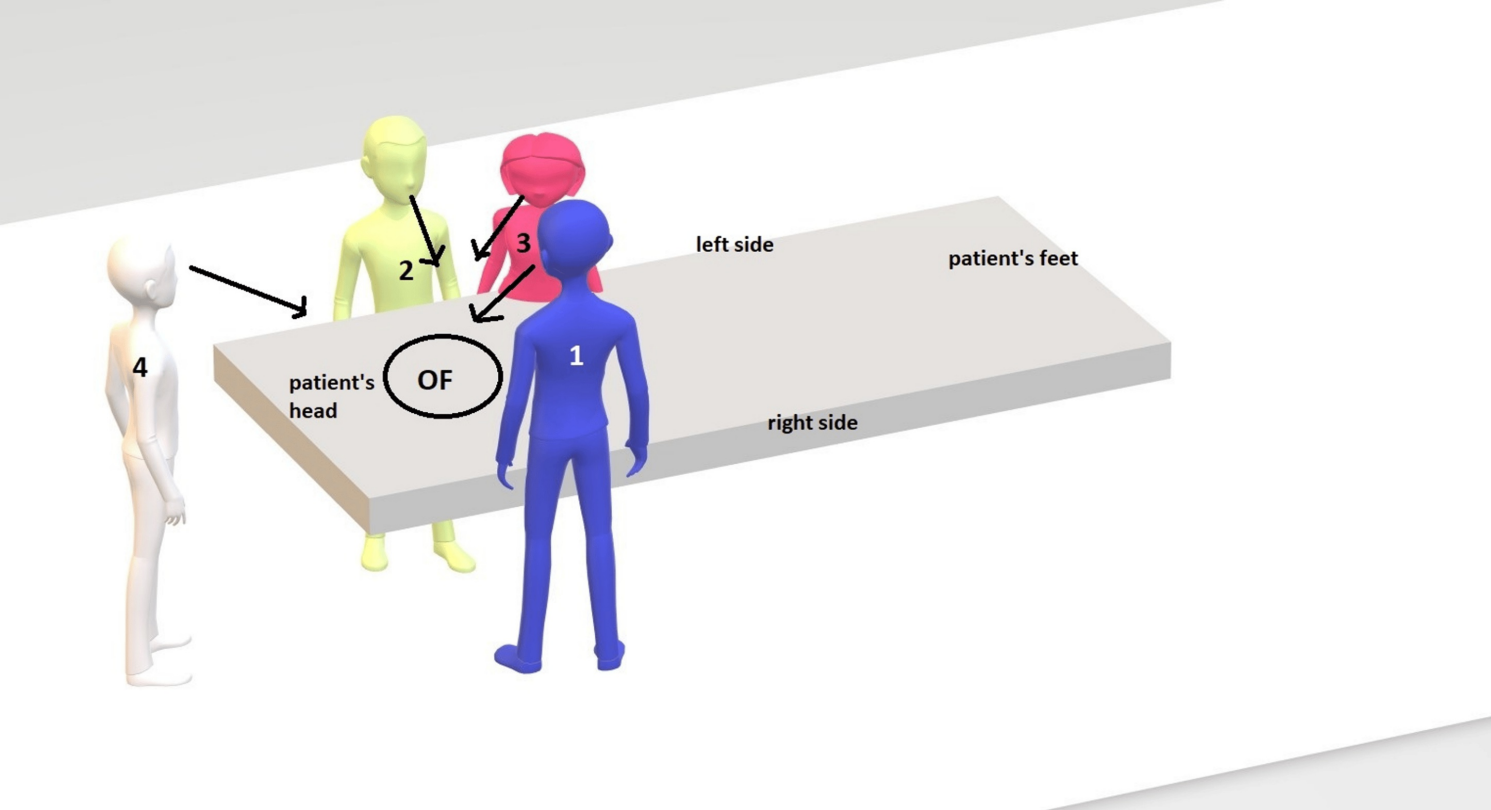
Optimal positioning of the surgical team during cardiac surgery, allowing direct visual access to the OF. 1: primary surgeon; 2: assistant surgeon; 3: scrub nurse; 4: anesthetist; OF: operation field Image credits: Dr. Idriss Souko.

Positioning the scrub nurse on the patient's left side provides a clear, unobstructed view of the operative field, unlike the conventional right-side layout, where the nurse often stands beside or slightly behind the surgeon with limited visual access. This visibility enables the nurse to anticipate instrument needs and respond to subtle nonverbal cues such as hand gestures, gauze requests, or upcoming suture steps (Figure 1).

As Catchpole et al. emphasize in their research on OR safety, disruptions in visual communication are significant contributors to miscommunication and delays during surgery, particularly in high-acuity specialties like cardiac surgery. They highlight that continuous shared visibility between team members reduces the cognitive burden of verbal clarification and enhances efficiency [1].

Shortened hand-off pathway and fewer adjustments

From an ergonomic standpoint, a scrub nurse stationed on the patient’s left can pass instruments across the midline with shorter, more intuitive hand movements, reducing the risk of contamination, tool dropping, or incorrect orientation. This is especially critical when handling delicate instruments such as valve sizers, coronary shunts, or mechanical valves. In contrast, when the nurse lacks a clear view of the field, instruments are often passed incorrectly, requiring rotation or repositioning by the surgeon, leading to delays and added frustration.

Matern and Koneczny note in their comprehensive report on OR design that instrument transfer ergonomics and spatial reach significantly affect operation time and surgeon satisfaction. Optimizing instrument trajectory reduces friction and delays during high-frequency exchanges [2].

Improved communication and anticipation

Cardiac operations depend heavily on precise team coordination and timing. A scrub nurse with direct visibility of the operative field is better equipped to act preemptively rather than reactively. According to Russ et al., anticipation of the surgeon’s needs is directly linked to visual access and spatial proximity. In their observational study across several cardiac ORs, nurses with direct visual access predicted more requests without verbal prompting [3].

Enhanced coordination with the assistant surgeon

The assistant surgeon, typically positioned on the patient’s left side, plays a critical role in suctioning, retracting, and tying sutures. When the scrub nurse is also stationed on the left, direct coordination with the assistant becomes possible eliminating the need to pass behind the primary surgeon or interfere with the surgical field. This triangle of efficiency, assistant, nurse, and field, proves especially effective during tasks such as pledget placement, knot tying, or suction hand-offs during bleeding control.

Personal experience: two setups, one clear preference

Having worked in two hospitals with distinct OR configurations, I have experienced firsthand the operational impact of scrub nurse positioning. In the first hospital, the scrub nurse was consistently placed on the patient’s left side, directly opposite me. In the second, the nurse stood beside me on the right, often partially obstructed and without a full view of the surgical field.

The contrast was striking. In the first setup, hand-offs were smoother, anticipation was sharper, and workflow was more fluid. The nurse required fewer verbal prompts. This alignment enhanced efficiency and reduced mental fatigue, an essential factor in long, complex operations.

While the principles of ergonomics and human factors strongly support positioning the scrub nurse on the patient’s left side, real-world experience further validates these findings. From enhanced visibility and communication to reduced hand-off delays and improved team coordination, the benefits are practical and measurable. Ultimately, personal preference and team dynamics will continue to play a role. Nevertheless, as surgical teams strive for greater efficiency and safety in cardiac procedures, reevaluating the scrub nurse’s position should be a key consideration in OR design discussions.
